# Physicochemical, Mineral and Sensory Characteristics of Instant *Citrullus lanatus mucosospermus (Egusi)* Soup

**DOI:** 10.3390/foods10081817

**Published:** 2021-08-06

**Authors:** Olakunbi Olubi, Joseline Veronica Felix-Minnaar, Victoria Adaora Jideani

**Affiliations:** Department of Food Science and Technology, Cape Peninsula University of Technology, P.O. Box 1906, Bellville 7535, South Africa; olubio@cput.ac.za (O.O.); felixminnaarj@cput.ac.za (J.V.F.-M.)

**Keywords:** sensory evaluation, egusi, soup: nutritional, acceptability, mixture design, consumer

## Abstract

Defatted egusi flour offers a food option high in protein and essential micronutrients. An instant processing method was adopted in a ready-to-eat instant soup using egusi grit, hydrocolloid, and defatted flour. A D-optimal quadratic mixture model was used to study the effect of the independent variables (grit, flour, and hydrocolloid) qualities. The quadratic model was adequate to navigate the design space for taste and appearance. The numerical optimization for appearance and taste of instant soup (IES) was used to obtain the optimal soup mix of 10 g of hydrocolloid, 57.2 of defatted flour and 17 g of grits. Sixteen trace and five major mineral elements were found in the egusi soup, with a high concentration of phosphorus (1220.4, 1326.2 and 1277.9 mg/100 g), potassium (1220.4, 1326.2 and 1277.9 mg/100 g), magnesium (822.2, 905.3 and 863.70 mg/100 g), calcium (172.3, 190.9 and 183.4 mg/100 g) and iron (53.7, 57.5 and 29.5 mg/100 g), and for instant egusi soups from boiled egusi grit (IESBG), instant egusi grit from spherified grit (IESSG) and instant egusi grit from extruded grit (IESEG), respectively. The amino acid profile of instant egusi soup offers all essential amino acids necessary to nourish the body. Phosphorus content was significantly (*p* ≤ 0.05) high across the three soups: 1742, 1836 and 1838 mg/100 g for IESBG, IESSG, and IESEG, respectively; IESSG and IESEG were significantly (*p* ≤ 0.05) higher in minerals when compared with IESBG. Instant egusi soup differed significantly (*p* ≤ 0.05) in lightness (L*), while the redness (a*) and yellowness (b*) did not vary significantly.

## 1. Introduction

The seed of *Citrullus lanatus* subsp *mucosospermus* (egusi) is high in linoleic fatty acids, with a good amount of essential amino acid. This species of melon originates from western Africa, belonging to the cucurbit family. Egusi is rich in oils, proteins 60% *w*/*w*, fibre, ash, carbohydrate, and minerals. Consequently, egusi seed has long been domesticated and must be explored further. Egusi melon plant thrives in tropical weather, with complete maturity and cultivation expected after three months of planting [1]. Several authors have adopted several methods of processing to determine the best possible usage [2]. The traditional method of processing raw egusi flour is laborious and negatively influences its consumption. Therefore, there is a need to increase the accessibility of nutritious underutilized seeds to explore their benefits to a world suffering from poverty and malnutrition. Food processing improves food flavour and palatability by increasing the bioavailability of food nutrients. This improvement is achieved by inactivating anti-nutritional factors [3]. Precooking defatted egusi flour by conventional boiling, chemical cooking (spherification) and extrusion cooking into grits reduce the laborious cooking process of soup made from egusi meal [3]. Conventional boiling results to some physical and chemical changes in the component of egusi flour [4]. Boiling technology provides high-quality food products with enhanced flavour, colour, and biological and active components [4]. Boiling egusi flour in water and subsequently drying it to form grits will offer a precooked grit with an improved functional property. Even more so is the process of producing egusi grit with chemical cooking (spherification), which is an old technique in the world of modernist cuisine, and was pioneered at El Bulli in 2003, a cornerstone in experimental kitchens around the globe [4,5].

Spherification is the culinary process of shaping a liquid into spheres that visually and texturally has a moist inner texture [6]. In modern cuisine, this technique is central to forming faux caviar, eggs, gnocchi, and ravioli [4,5,6]. Although alginate gels allow gel formation, alginates and a calcium source were introduced in spherification technology in 2003 and have been around for decades in the food industry [7]. The first use of alginates was to restructure red peppers for manufacturing pimentos in olives. Unlike most edible gels, which are stable throughout, alginate spheres typically contain a physical outer gel membrane with a liquid core [7]. Spherification can occur using the basic or the reverse technique [8]. The reverse method utilizes a calcium source added to the edible liquid, broth or slurry and is dispensed into a sodium alginate bath. The reverse spherification explored in this study involves calcium (Ca^2+^) first cross-linking at the film surface, drawing the polymer chains closer together. This results in the formation of a less permeable surface, slowing the diffusion of Ca^2+^ [9,10,11]. Another possible method for producing precooked grits is extrusion cooking.

Extrusion is the modern pre-heat cooking technique using a single screw and mechanical shearing at relatively low moisture content levels [12,13]. Extrusion allows starch gelatinization, the denaturation of protein, microbial reduction, enzyme inactivation, and colour changes, the extent of which is dependent on the conditions of the extrusion. Those changes at the constituents’ level modify the rheological behaviour of flour batters. Extrusion cooking is also responsible for changing molecular associations between components such as the amylose–lipid complex that can affect the in vitro starch digestibility of the flours [14,15].

Egusi seed is locally defatted by cold pressing by hand and is predominantly consumed in western, central and eastern Africa as a local relish called egusi/melon soup [16]. Egusi soup is widely known for its characteristics, taste, and flavour, consumed for decades without a formal introduction into the food industry. The laborious method of its preparation has been a significant deterrent to its consumption. A widely known instant soup is the traditional freeze-dried instant vegetable soups in the global market. It is often prepared with various vegetables as primary raw materials, seasoning, and freeze drying [17].

Freeze drying produces the highest quality of dried foods. However, a significant problem with conventional freeze drying is the long drying time needed, leading to high energy consumption and capital costs [17]. The use of freeze-drying methods is partly due to the poor heat transfer associated with the conventional electric heating method, which transfers heat for drying by conduction [17]. A high cost of production restricts the popularity of freeze-dried instant soups. Thus, an instant cook mix that involves less processing and low energy utilization was explored in this study.

An instant cook mix drinking soup from egusi has never been explored or industrialized. The ease of its preparation will fit into the busy life of an average soup eater, thereby offering a nutritionally dense soup. The continuous yearn for modern life and the increase in the number of people depending on fast food has resulted in food preparation and consumption habits requiring less time for cooking a healthy meal [17]. According to the report “Specialty Food Consumer 2010” by the National Association for the Specialty Food Trade (NASFT), there is a growing consumer preference for eating at home [17]. Along with the improving economic conditions, a surprising number of consumers had re-engaged with speciality foods in 2010 [18]. Speciality meals, such as egusi instant soup, are generally easier to handle over time-consuming conventional cooking [8], while still offering daily nutrient intake, even more so during COVID-19 global pandemic and emergence of chronic diseases. A meal high in essential nutrients will promote healing and enable the body to fight for optimum health [19]. Therefore, the objectives of this study were to produce an optimal instant drinking egusi soup and determine its physicochemical and sensory properties.

## 2. Materials and Methods

### 2.1. Source of Egusi Seed, Chemical, and Equipment

All laboratory chemicals and food-grade reagents were obtained from Merck Pty, South Africa. Egusi oil was obtained from oil extracted by supercritical CO_2_ extraction. Egusi hydrocolloid and defatted egusi flour were obtained from processed defatted egusi flour and hydrocolloid. The soup spice was obtained from GR Spice, Cape Town, South Africa.

#### 2.1.1. Production of Defatted Egusi Flour by Supercritical CO_2_ Extraction

Defatted egusi flour was obtained after supercritical fluid extraction using three experimental runs. These were conducted using a low temperature, low pressure (60 °C, 450 bar), low temperature, high pressure (55 °C, 600 bar) and high temperature, high pressure (75 °C, 600 bar). The extractor was loaded with approximately 2000 g of raw dehulled egusi seed in each experiment. Firstly, the system was pressurized (at intervals of approximately 400–600 bars) until the desired pressure of 450 bar and 600 bar was achieved. After the column temperature and the pressure were stabilized and kept in contact with the egusi seed for at least 15 min to allow system stabilization, the supercritical CO_2_ was pumped into the bed of the egusi seed at a constant 30 g/h using the expansion valve at the outlet of the extractor. At the extraction temperature between 55, 60 and 75 °C, a thermoregulatory device allowed the separation between the extract and the solvent during depressurization. The defatted egusi flour (DEF) was collected via a glass container and weighed every 10–20 min until a constant weight was determined. Defatted egusi flour was tested for its nutritional and functional properties.

#### 2.1.2. Production of Instant Egusi Grits from Defatted Egusi Flour Using Different Processing Methods

Defatted egusi flour was subjected to various processing techniques to precook the flour into grits. These processing techniques include boiling, spherification, and extrusion.

#### 2.1.3. Production of Instant Egusi Grits Using the Boiling Method

Defatted egusi flour (800 g) was weighed into a bowl, mixed with 200 g of water to form a thick paste, cut into small balls (1 cm in diameter), and placed in boiling water to boil for 5 min. The cooked balls were removed from the boiling water and dried with an electric cabinet drier at 50 °C for 24 h. The dried egusi crumbs/balls were milled with a Corona hand mill (Landers, Corona Mill, Columbia) and sieved to pass through a 355 µ mesh. Boiled egusi grits were then stored in air-tight plastic containers and held at 4 °C until further use.

#### 2.1.4. Production of Instant Egusi Grits Using Spherification

Egusi slurry was prepared by mixing defatted egusi flour with water in a 1:1.5 g/mL ratio to form a thick slurry. To 250 g of egusi slurry, 5 g of food-grade calcium lactate was added to increase its calcium content. The sodium alginate bath was prepared by adding 5 g of sodium alginate in 1000 g of distilled water and mixing with an electric blender for 5 min. The mixture was allowed to hydrate for 24 h in the refrigerator to remove the bubbles formed during blending. Egusi slurry enriched with calcium lactate was filled into a syringe, and 0.1 mL was dropped into the alginate bath. The balls were taken out from the bath with a spoon, rinsed twice with distilled water and dried in a cabinet drier at 50 °C for 24 h. The dried flakes were milled with a Corona hand mill and sieved through a 355-micron sieve. The spherified grits were then stored in air-tight plastic containers and held at 4 °C until further use.

#### 2.1.5. Production of Instant Egusi Grits Using Extrusion

Defatted egusi flour (DEF) was subjected to manual extrusion utilizing a pasta extruder (Automatic pasta maker OTTIMO, Gauteng, South Africa). Defatted egusi flour and water ratio 2.5:1 g/mL was used during the extrusion. Defatted egusi flour of 250 g and 100 mL of water was weighed into the mixing chamber of the extruder, the chamber was closed, and the “mix” button was pressed to initiate the mixing process. After mixing for 2 min, the “extrude” button was pressed to extrude the mixed egusi. Extruded egusi was dried at 50 °C for 24 h in a cabinet dryer and then milled with a Corona hand mill and sieved with a 355-micron sieve. Extruded grits were then stored in air-tight plastic containers and held at 4 °C until further use.

### 2.2. Optimization of Instant Soup Mix Using Mixture Design

A D-optimal mixture design was used to determine the effect of boiled egusi grit (*X*_1_), defatted egusi flour (*X*_2_) and egusi hydrocolloid (*X*_3_) on the sensory liking of instant egusi soup. The three components amounted to 85% *w*/*w* of the mix, comprising 15 to 20% for egusi grits, 55 to 60% *w*/*w* for egusi flour, and 5 to 10% *w*/*w* for egusi hydrocolloid. The design consisted of 11 formulations with 5 vertices, 1 centre, 3 centre edges and 2 axial points (Table 1). The instant soup was prepared by mixing the three components outlined for each formulation in Table 1 in a random order and mixing with egusi oil (10%) and spices (5%). The soup mix was combined and dispersed in 61.4 mL/100 g of boiled water at 100 °C, cooked for 5 min, and served to panellists.

### 2.3. Benchtop Sensory Evaluation of the Soup Mixes and Data Analysis

Five consumer panellists comprising one male and four females were chosen among the Cape Peninsula University of Technology staff. The age of the panellists ranged between 25–40 years. The 11 soup mixes from boiled grits were used to optimize the mix and were prepared into instant soup by adding boiling water (61.4 mL/100 g of soup mix) and mixed for 5 min. The panellists received prior information about the procedure and were requested to sign an informed consent form before tasting the products. Three-digit coded samples in a randomized order were presented to each panellist with a cup of water to rinse their palate between tasting each sample. They were instructed to rate each soup for appearance, colour, aroma, taste, and overall acceptability using a five-point hedonic scale as (1) dislike extremely, (2) dislike moderately, (3) neither like nor dislike, (4) like moderately, (5) like extremely.

The sensory data were modelled using a quadratic mixture model (Equation (1)) to establish the relationship between the sensory response variables and the three components of the instant soup [20].

(1) $$Y=\beta_{1}X_{1}+\beta_{2}X_{2}+\beta_{3}X_{3}+\beta_{12}X_{1}X_{2}+\beta_{13}X_{1}X_{3}+\beta_{23}X_{2}X_{3}$$

where *Y* represents the predicted sensory attribute: *β*_1_, *β*_2_, and *β*_3_ represent the expected response from a pure mixture with *X*_1_ = 1 (and all other components zero), egusi grit, egusi flour, and egusi hydrocolloid, respectively. The component proportion or independent variables *X*_1_, *X*_2_ and *X*_3_ refer to the egusi grit, egusi flour and hydrocolloid, respectively; *β*_i_ is the linear blending term. *Β*_ij_ is the nonlinear blending term which when it is positive, the term is synergistic, and if negative, it is antagonistic.

Analysis of variance (ANOVA) was used to establish the statistical significance for each independent variable on the sensory attributes. The goodness of the model fit was determined by the lack of fit, adequate precision, and residual plots. The Trace peipel plot plots were used to interpret the effects of the mixture components on the sensory attributes [20]. Mixture models that adequately explained the variability in the data were used to search for the optimum mixture for the soup mix.

### 2.4. Optimal Soup Mix from the Quadratic Mixture Model and Instant Egusi Soup Production

Numerical optimization was used to obtain an optimal mixture of egusi grits, egusi flour and egusi hydrocolloid based on having the components in range while maximizing the appearance and taste of the instant egusi soup. 

The optimum formulation was then used to produce three soups using the three grits (boiled, spherified, and extruded) by adding appropriate amount of water. The optimum soups from the three grits were subjected to physicochemical, microbial, and consumer liking tests.

### 2.5. Colour Measurement of Instant Egusi Soup

The colour of the instant soup mixes was determined using a Minolta Chroma Meter (CR300) [21] using a CIE standard illuminant D65 and 10° observer to determine CIE colour space coordinates, L*a*b*. The instrument was calibrated using a white calibration tile CR-A44. The L* measures the degree of whiteness/darkness, and the higher the L* value, the higher the whiteness intensity. The a* indicates the balance between redness and greenness of the sample, with a positive value corresponding to redness and a negative value to greenness. In contrast, the b* value shows the balance between yellowness (+) and blueness (−). The values closer to zero indicate less intense colour for a* and b* readings, whereas reading further from zero corresponds to more intense chroma characteristics [22]. The readings were collected in triplicate.

The chroma and hue were calculated as shown in Equations (2) and (3).

(2) $$C=\sqrt{a^{*2}{+b}^{*2}}$$

where C = chroma; a* = redness/greenness; b* = yellowness/blueness.

(3) $$H=\tan^{-1}[\frac{b^{*}}{a^{*}}]$$

where H = hue angle; a* = redness/greenness; b* = yellowness/blueness.

### 2.6. Mineral Content of Instant Egusi Soup

The mineral content of egusi soup was carried out according to the standard AOAC official method [23]. First, samples were prepared by solubilizing the acid-extractable elemental material. Then, digestion was performed on a microwave digester, using ultra-pure HNO_3_, at an elevated temperature and pressure. After a cooling period, the extractant was made up to a 50 mL volume with deionized water, then analyzed by an Inductive Coupled Plasma Mass Spectrophotometer (ICP-MS) or Inductive Coupled Plasma Atomic Emission Spectroscopy for the significant mineral analytes.

Trace elements were analyzed on an Agilent 7700 quadruple ICP-MS. Samples were introduced via a 0.4 mL/min micro-mist nebulizer into a Peltier-cooled spray chamber at a temperature of 2 °C, with a carrier gas flow of 1.05 L/min. The instrument was calibrated using NIST (National Institute of Standards and Technology, Gaithersburg, MD, USA) traceable standards to quantify selected elements. The sample underwent a digestion step, and the results were corrected for the dilution factor resulting from the digestion procedure.

Major mineral elements (Na, K, Ca, Mg and P) were analyzed on a Thermo ICap 6200 ICP-AES. The instrument was calibrated using NIST (National Institute of Standards and Technology, Gaithersburg, MD, USA) traceable standards purchased from Inorganic Ventures (Inorganic Ventures 300 Technology Drive, Christiansburg, VA 24073, USA) to quantify selected elements. In addition, NIST traceable quality control standards, from De Bruyn Spectroscopic Solutions, Bryanston, South Africa, were analyzed to verify the calibration accuracy before sample analysis and throughout the analysis to monitor drift. The instant soup samples were analyzed in triplicate.

### 2.7. Microbiological Evaluation of Instant Egusi Soup

Samples of instant egusi soup (10 g) were dispersed in 90 mL sterile Ringer’s solutions, using ¼ strength Ringer’s solution (Merck, Darmstadt, Germany) under the aseptic conditions. One tablet (BR001) in 500 mL of distilled or deionized water was stirred slowly until complete dissolution. The solution was dispensed in tubes (9 mL) and sterilized in an autoclave at 121 °C for 15 min. The solution was cooled to 25 °C after incubation.

A portion (10 g) of the instant egusi soup was aseptically placed into a sterilized bag (10–15 cm, Sunkyung Co., Seoul, Korea) with 90 mL of sterile peptone water (0.1%) N. The mixture was homogenized in a stomacher blender (Model 400, Teledyne Tekmar Co., Mason, OH, USA) for 2 min. The following mediums were used for culturing: plate count agar (Difco Co., BD Diagnostic Systems, Sparks, MD, USA) for total aerobic bacteria, and 3M Petrifilm (3M Health Care, St. Paul, MN, USA) for coliform. A 1 mL aliquot was spread onto plates containing one of the media and incubated for bacterial growth at 35 °C for 48 h under aerobic conditions. Microbial populations from the sample cultured in triplicate on each medium were evaluated manually, counting the colonies using a colony counter for each plate [24]. The total count of mesophilic aerobic bacteria (TMAB) was enumerated in pour-plates of plate count agar (Merck) after incubation at 30 °C for 48 h. Yeast and moulds were identified in potato dextrose agar (Merck) agar plates after incubation at 30 °C for three days.

### 2.8. Sensory Evaluation of Instant Egusi Soup

A consumer tasting panel of 50 was invited among Cape Peninsula University of Technology staff and students to determine instant egusi soup sensory quality attributes. The demography of the 50 consumer panellists showed that 86.0% were female, 14.0% were male, 94.0% were students (52.0% local students, 48.0% international students), 6.0% were staff, 90% were in the <20–29 age group, 8.0% were in the 30–39 age group, and 2.0% were in the 40 and above age group. The black race comprised 92.0%, the coloured race 6.0%, and the white race 2.0% of the panellists. Panellists received written and prior verbal instruction regarding the evaluation procedures. Each panellist was presented with three three-digit coded 30 mL instant egusi soup in a randomized order in foam cups on a 9.6 × 6.6 cm tray and water to rinse their palate between tasting. They were instructed to rate each sample for appearance, colour, aroma, taste, texture, and overall acceptability using a five-point hedonic scale (1) dislike extremely, (2) dislike moderately, (3) neither like nor dislike, (4) like moderately and (5) like extremely.

### 2.9. Statistical Data Analysis

All data were expressed as the mean ± standard deviation of triplicate readings. The data, including the consumer sensory, followed a normal distribution and were subjected to a multivariate analysis of variance to establish the mean differences between treatments. Duncan multiple range tests were used to separate means where differences existed at *p* = 0.05 (IBM SPSS Statistics version 24, IBM Corporation, Chicago, IL, USA).

## 3. Results

### 3.1. Production of Instant Egusi Grits

**Figure 1:**
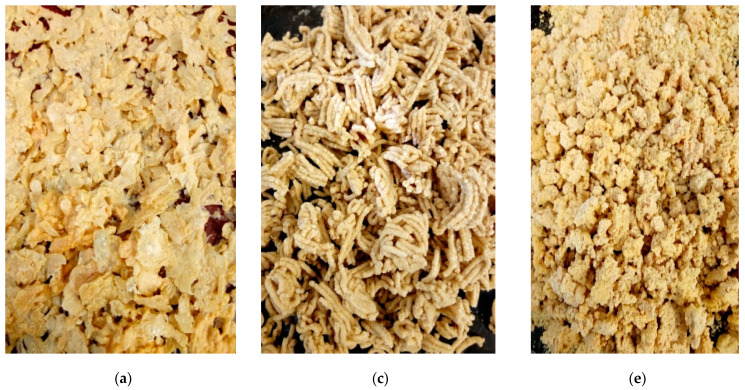

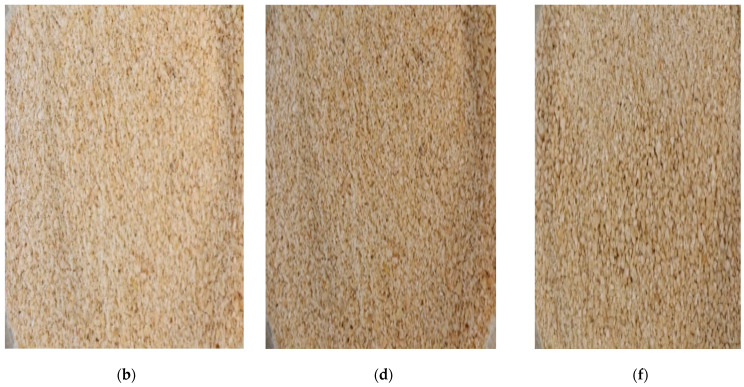
(**a**) Spherified egusi grit unmilled; (**b**) spherified egusi grit milled; (**c**) extruded egusi grit unmilled; (**d**) extruded egusi grit milled; (**e**) boiled egusi grit unmilled; (**f**) boiled egusi grit milled.

Precooked grits were made from defatted egusi flour, using three different processing methods: boiling, spherification, and extrusion cooking, as shown in Figure 1. The boiled grits formed coarse crumbs after drying. Both boiled and extruded grits were pale yellow. Egusi grit made from reverse spherification formed a flaky irregular shape after drying. The flakiness of the spherified egusi grit could be due to the extra film formed by the alginate gum.

### 3.2. Optimization of Instant Egusi Soup

#### Model Fitting

The sensory properties of instant egusi soup (IES) recorded in Table 2 shows the mean and standard deviation of the 11 instant egusi soup formulations made from the boiled grit. The regression coefficient of the quadratic mixture model for instant egusi soup (IES) is shown in Table 3.

The high regression coefficient for the variables, except for aroma, suggests that the quadratic mixture model explains the variability in the sensory data. There was a non-significant lack of fit for each model with a model adequate precision of greater than 4, except for aroma, indicating an adequate signal. A negative pred R^2^ (−4.62) implies that the overall mean may be a better predictor of the response on aroma than the quadratic model. However, Table 4 indicates that the quadratic model was only significant (*p* ≤ 0.05) for appearance and taste. Hence, the quadratic model can be used to navigate the design space for appearance and taste.

## 4. Discussions

### 4.1. Effect of Mixture Component on the Instant Soup Sensory Parameters

The linear effect of the component did not significantly affect the sensory attribute of the instant egusi soup shown in Table 4. The quadratic blend of egusi grit and egusi flour (*X*_1_*X*_2_) (egusi grit and egusi hydrocolloid) had a significant (*p* ≤ 0.05) synergy effect on appearance and taste. The relationships are shown in Equations (4) and (5)

(4) $$\mathrm{Appearance}=3.26X_{1}+3.34X_{2}+3.28X_{3}+2.78X_{1}X_{2}+1.94X_{1}X_{3}-0.22X_{2}X_{3}$$

(5) $$\mathrm{Taste}=3.00X_{1}+3.50X_{2}+2.80X_{3}+1.86X_{1}X_{2}+3.76X_{1}X_{3}+0.13X_{2}X_{3}$$

where *X*_1_ = boiled egusi grit; *X*_2_ = egusi flour; *X*_3_ = egusi hydrocolloid.

**Figure 2:**
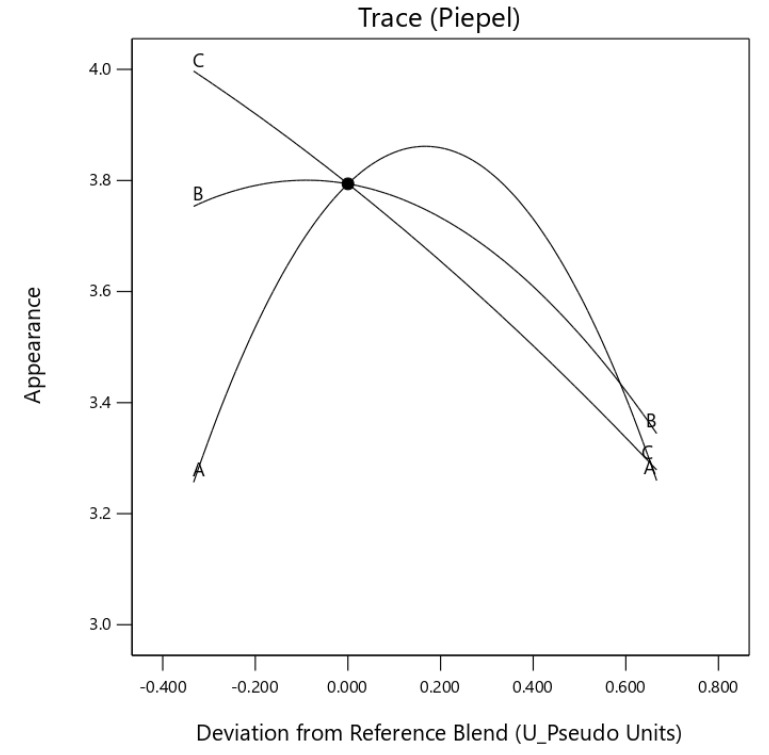
Trace (Piepel) plot for the effect of three components on appearance (A: boiled egusi grit, B: flour and C: hydrocolloid) on instant egusi soup.

The trace piepel plot (Figure 2) showed the quadratic curve was exerting a positive influence resulting in a concave curve for appearance. Egusi grit and egusi flour (*X*_1_*X*_3_) also had a synergic effect on the appearance of IES. The taste of IES also had a synergistic effect as egusi grit and egusi flour (*X*_1_*X*_2_) was increased in the instant soup, with the vertex edges indicating the impact of each ingredient.

**Figure 3:**
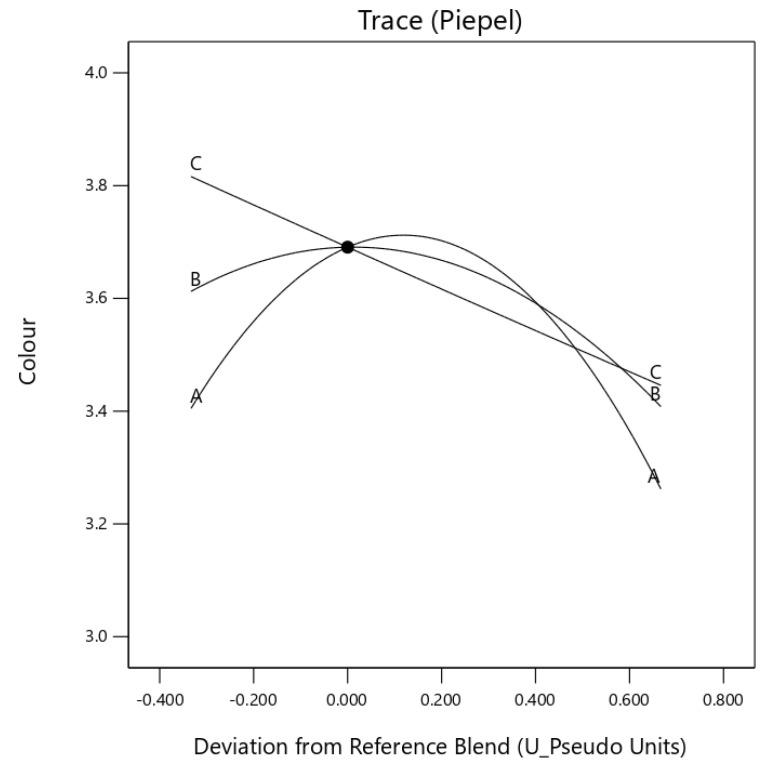
Trace (Piepel) plot for the effect of three components on colour (A: boiled egusi grit, B: flour and C: hydrocolloid) on instant egusi soup.

**Figure 4:**
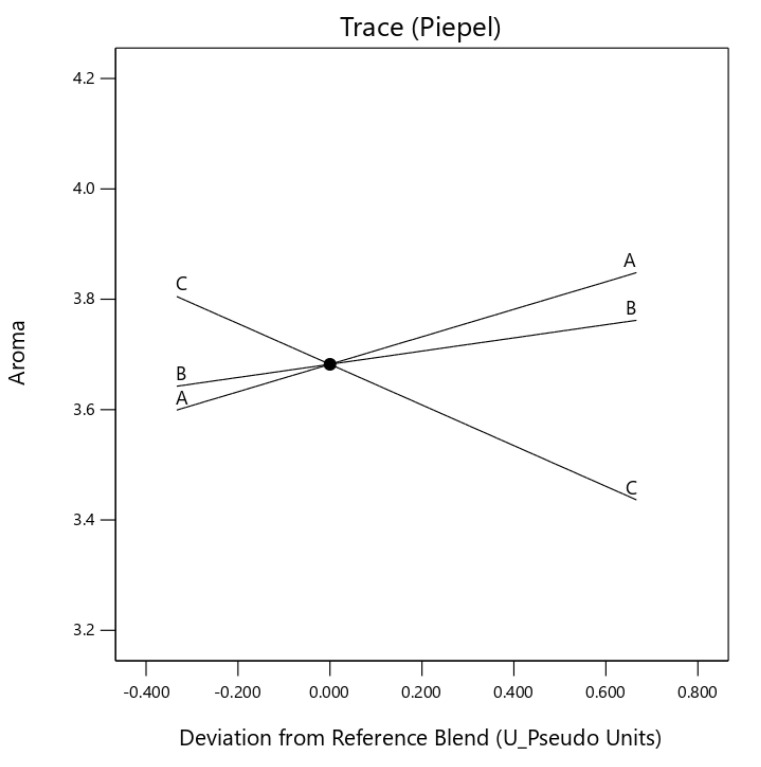
Trace (Piepel) plot for the effect of three components on aroma (A: boiled egusi grit, B: flour and C hydrocolloid) on instant egusi soup.

**Figure 5:**
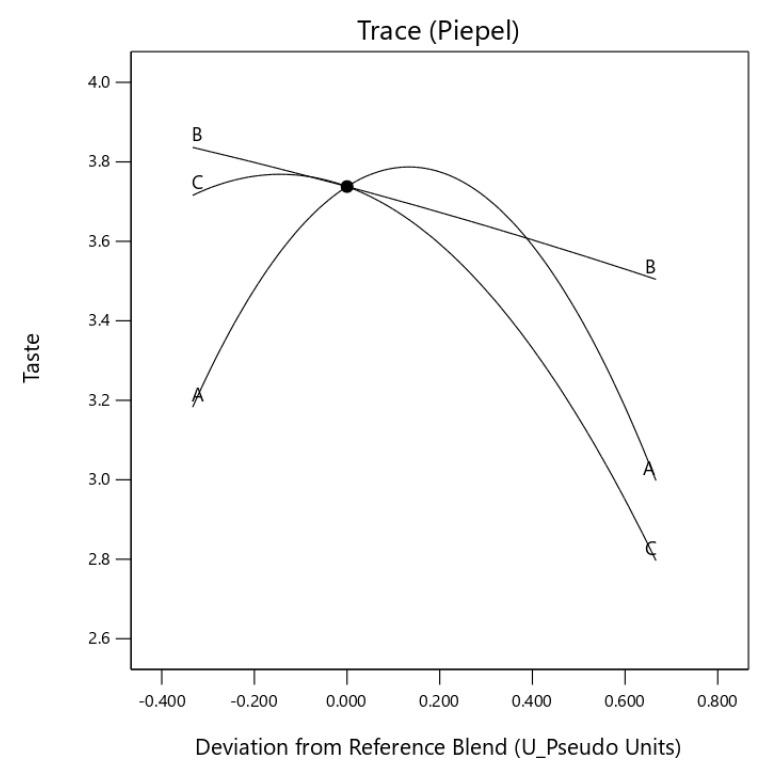
Trace (Piepel) plot for the effect of three components on taste (A: boiled egusi grit, B: flour and C hydrocolloid) on instant egusi soup.

**Figure 6:**
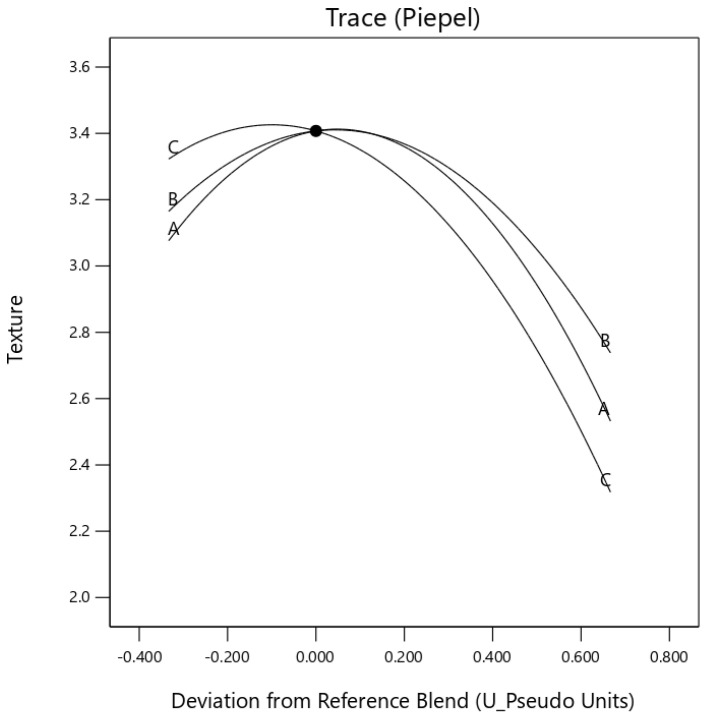
Trace (Piepel) plot for the effect of three components on texture (A: boiled egusi grit, B: flour and C hydrocolloid) on instant egusi soup.

Component egusi grit and egusi hydrocolloid (*X*_1_*X*_3_) and egusi flour and egusi hydrocolloid (*X*_2_*X*_3_) showed an antagonistic effect on the colour and aroma of the instant soup (Figure 3 and Figure 4). A significant (*p* ≤ 0.05) synergic effect was also observed for the taste of IES, for *X*_I_*X*_2_ and *X*_2_*X*_3_, displaying with a concave curve for the effect Figure 5. The component mix effect on the texture of instant egusi soup showed an antagonistic effect as seen in Figure 6.

**Figure 7:**
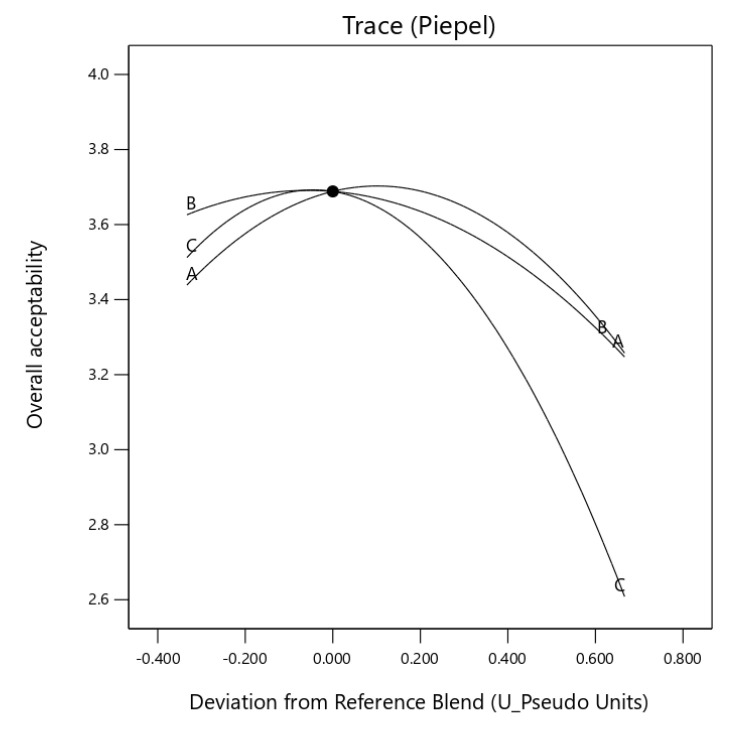
Trace (Piepel) plot for the effect of three components on overall acceptability (A: boiled egusi grit, B: flour and C hydrocolloid) on instant egusi soup.

The trace piepel plot also confirmed the non-significant effect of the increased addition of mixture components. A significant (*p* ≤ 0.05) synergic effect was observed with the component mix of *X*_1_*X*_3_ (egusi grit and hydrocolloid) for the overall acceptability IES. The mix of *X*_1_*X*_2_ and *X*_2_*X*_3_ indicated an antagonistic effect of the component mix, as showed in Figure 7.

#### Optimal Instant Egusi Soup Mix

The optimization goal was to have the components (A (boiled egusi grit), B (egusi flour) and C (egusi hydrocolloid)) in range while maximizing appearance and taste. The optimal egusi soup mix with the desirability of 0.947 was predicted as 57.2% defatted egusi flour, 17% boiled egusi grits and 10% egusi hydrocolloid. 

### 4.2. Colour of Instant Egusi Soup

Table 5 shows that the CIELab colour parameters for instant egusi soup (IES) changed after processing. The values for the L*, a* and b* coordinates of the instant soup made from boiled grit (IESBG) were 63.47, 5.77 and 18.15, respectively. Due to Maillard’s reaction, the boiled egusi grit, cooked at boiling temperature, showed a creamy white colour. A Maillard reaction is a chemical reaction between amino acids and reducing sugars that gives browned food its distinctive flavour.

After processing, a colour change was observed, resulting in the low redness of instant egusi soup made from boiled grit. A similar report was given by Akobundu [24], who reported that the colour of the egusi meal changes to a pale brown colour when cooked, which invariably is responsible for the pale colour of sausages made using egusi meal. The coordinates for instant egusi soup made with spherified grit (IESSG) were 63.32, 6.00, and 17.75, respectively, in Table 5. At the same time, the L*, a* and b* coordinate of instant egusi soup made with extruded grit (IESEG) were 62.89, 6.34, and 17.56, which differed significantly (*p* ≤ 0.05) in lightness L from IESBG and IESSG. Lightness in the colour of boiled and spherified instant soup could be due to the breakdown of starch granules during extrusion, thereby causing more water absorption. The chroma and hue angle results showed colour stability for the instant soup samples shifting towards the yellow region [25]. The hue angle of fresh egusi soup was about 72 °C, representing a colour in the yellow region (hue angle between 0° and 90°).

Comparing the three soup samples, it is possible to conclude that the soups were pale yellow and had no significant redness and yellowness. However, the lightness L* significantly differed in the soup made with extruded grit (IESEG), which could be due to extrusion on the protein and starch structure of the extruded grit, thereby causing more water absorption in the soup. The hue angle for IESBG, IESSG and IESEG were 72.0°, 71.3° and 70.1°, respectively, indicating the soups were within the light yellow.

### 4.3. Trace and Major Mineral Content of Instant Egusi Soup

Sixteen trace mineral elements and five major elements were detected in instant egusi soup, as shown in Table 6 and Table 7. The instant egusi soup (IES) produced with boiled (IESBG) grit, spherified grit (IESSG) and extruded grit IESEG) were high in major mineral elements (Table 6). Phosphorus was high in instant egusi soup: 1742.8, 1836.3, and 1838.2 mg/100 g for IESBG, IESSG, and IESEG, respectively, which differed significantly (*p* ≤ 0.05). This high phosphorus was not similar to the report made by Enujiugha and Ayodele-Oni [26]. It was reported that phosphorus (2.8 g/kg), magnesium (3.43 g/kg) and potassium (3.9 g/kg) is present in egusi melon, which is low to the proportion found in instant egusi soup.

The mineral content was significantly high in the soup made with spherified and extruded grit. This high mineral content could be due to the processing of the precooked grit. The spherified grit was obtained using reverse spherification techniques that involved calcium chloride and an alginate bath in forming the spherified ball, which was subsequently dried and milled into a precooked grit [26]. IESSG was significantly (*p* ≤ 0.05) high in minerals except for Fe (53.7, 57.5 and 29.5 mg/100 g) and Al (23.0, 15.6 and 10.6 mg/100 g), which was high in the instant soup made from boiled grit.

Being the highest in IES, phosphorus is essential in the human diet, as it helps combat diseases. In medicine, hypophosphatemia, a phosphate deficiency syndrome, may be caused by malnutrition, the non-absorption of phosphate, and metabolic problems resulting in acute illnesses. All causes of deficiency are associated with the low phosphate level in the blood serum and its cells. Symptoms of hypophosphatemia include neurological dysfunction and the disruption of muscle and blood cells due to a lack of ATP [27]. In addition, high phosphate intake can lead to diarrhoea, hardening of the organs, and soft tissue, a condition known as calcification. Calcification interferes with the body’s ability to digest iron, calcium, magnesium, and zinc [28]. In this view, instant egusi soup, being high in phosphorus, offers an instant functional meal [29].

The instant soup contains a high concentration of calcium: 172.3, 190.9, and 183.4 mg/100 g for IESBG, IESSG, and IESEG (Table 7), respectively, making it an alternative calcium source for a plant. In addition, the soups were also high in magnesium: 807.5, 880.1, and 957.8 mg/100 g, respectively, for IESBG, IESSG, and IESEG. Generally, the highest trace mineral present in instant egusi soup are Al (10.8–23 mg/100 g), B (2.8–3.1 mg/100g), Mn (7.7–10.5 mg/100 g) Zn (9.9–12.3 mg/100 g), and Si (11.5–17.0 mg/100 g). The major food sources of silicon were unrefined grains, cereal products, and root vegetables. Si is considered safe in food when present in a considerable amount.

Magnesium helps maintain healthy muscle and nerve functions that keep the heart rhythm steady. Calcium, potassium, and magnesium are needed to repair worn-out body cells and make red blood cells [30]. In addition, the three instant soups were sources of trace elements such as iron, zinc, and manganese. These elements are essential for enzyme metabolism and the proper functioning of individual cells in the body. The high phosphorus, magnesium, potassium, and calcium content in egusi soup make it an alternative healthy meal for pregnant lactating women, children, and older adults. The instant egusi soup contains a very low quantity of valium and boron, indicating that this flour could be a source of such elements required by tissue [30].

### 4.4. Microbiological Characteristics of Instant Egusi Soup

There were no aerobic bacteria detected and no cluster of colony growth seen after 48 h incubation. Coliform bacterial growth was not detected in the three instant soups. The total coliform bacteria are an indicator of the presence of pathogenic intestinal bacteria. Yeast and moulds were not detected after three days’ incubation, making instant egusi soup microbiologically safe.

### 4.5. Consumer Acceptability of the Egusi Soup

Egusi soup was produced with boiled, spherified and extruded grits based on 57.2% flour, 17% boiled egusi grit, 10% egusi hydrocolloid and 5% spice mix with 61.4 g/100 g egusi mix boiled water and 10% egusi oil and manually mixed for 5 min using a wooden spoon. The demography of the 50 consumer panellists, detailed in Table 8, shows 86.0% were females, 14.0% were males, 94.0% were students (52.0% local students, 48.0% international students), 6.0% were staff, 90% were in the <20–29 age group, 8.0% were in the 30–39 age group and 2.0% were in the 40 and above age group. The black race comprised 92.0%, coloured race 6.0% and white race 2.0% of the panellists (Table 8). Panellists’ response significantly (*p* ≤ 0.05) differed in the evaluation of the soup samples. However, there was no significant (*p* > 0.05) difference in the quality attributes of the soups.

The appearance of the instant egusi soup was neither liked nor disliked by the panellists: 3.0, 3.0 and 3.2, respectively, for IESBG, IESSG and IESEG. Based on various panellist comments, the soup appearance, taste, and colour would improve if the soup had a smooth texture. The grittiness of the soup was not welcomed by most panellists, which gave the soup a lower rating. Panellists gave the soups a positive comment in regard to the aroma, which included “nice aroma”, and “good aroma and good taste”. However, negative comments such as “bad appearance”, “I love the aroma not sure about the texture and colour”, were indicated.

**Figure 8:**
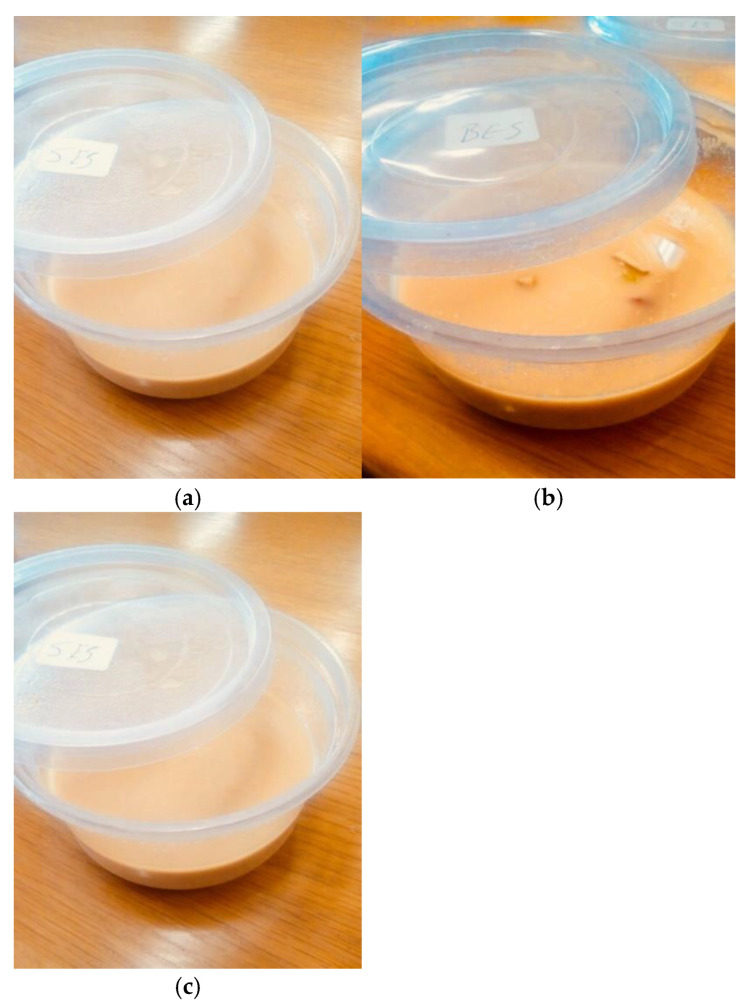
Instant egusi soup made from (**a**) boiled grits, (**b**) spherified grits and (**c**) extruded grits.

**Figure 9:**
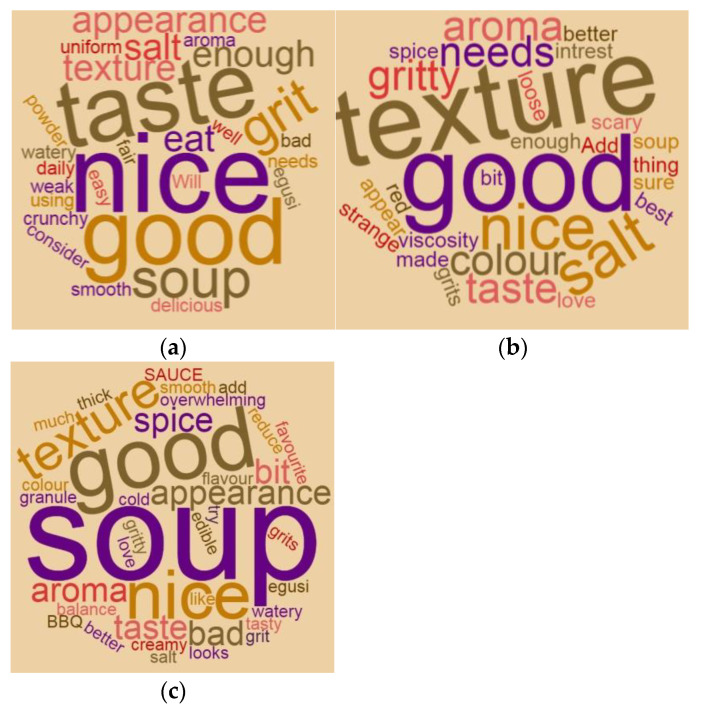
Sensory word cloud for instant egusi soup made with (**a**) boiled grit; (**b**) spherified grit (**c**) extruded grit.

The overall acceptability of instant egusi soup shown in Figure 8 ranged from 3.4–3.6 (liked moderately) for IESBG, IESSG and IESEG, with no significant difference among the three soup samples. Egusi instant soup with spherified and extruded grit had the best overall qualities based on all sensory attributes. According to the word cloud in Figure 9, boiled egusi soup has some negative comments: “soup is too weak”, “soup is too gritty”. It gained positive comments, such as “nice soup”, “good taste”, and “good soup”. These comments display the different options of each panellist, and therefore, instant egusi soup made from boiled grit could be accepted if subsequently improved. Egusi soup made from spherified grit gained primarily positive comments rather than negative. A few negative comments for IESSG were “bad appearance” and “watery soup”. This response confirms the diversity of each panellist, making instant egusi soup from spherified and extruded grits the most preferred soup among the soup samples. Egusi soup made with spherified grits gained positive comments, including “nice, good aroma, taste nice, good texture”, while a few negative comments were “gritty soup and bad texture”. These positive comments also revealed that the panellists moderately accepted the soup made from spherified grit and extruded grit (Figure 9).

## 5. Conclusions

Instant egusi soup produced with boiled, spherified and extruded grit offers a precooked meal that could fit into an average soup consumer’s daily busy meal schedule. The defatted flour used in the soup mix was high in protein, 60% *w*/*w* with a rich amino acid profile. The instant egusi soup is high in 21 trace and major minerals, with phosphorus (1838.2 mg/100 g), magnesium (905.3 mg/100 g), and potassium (1326.2 mg/100 g) being the highest mineral in instant egusi soup. The panellists moderately liked instant egusi soup. The overall acceptance for the soups was in the order of spherified ˃ extruded ˃ boiled. Thus, the objective of producing instant egusi soup with egusi grits was achieved. Instant egusi soup can be offered as an immediately accessible nutrition option to replenish health and vitality, especially in a world where immune-boosting has become an essential prerequisite to optimum health. A further improvement to the taste and texture must be explored to improve its sensory attribute in future studies.

## Figures and Tables

**Table 1:** D-optimal mixture design for instant egusi soup formulations.

	Independent Variables (%)		
Formulation	Boiled Grit	Flour	Hydrocolloid
1	19.2	56.7	9.2
2	15.0	60.0	10.0
3	20.0	55.0	10.0
4	20.0	60.0	5.0
5	18.3	58.3	8.3
6	17.5	57.5	10.0
7	17.5	60.0	7.5
8	20.0	55.0	10.0
9	15.0	60.0	10.0
10	19.2	59.2	6.7
11	20.0	57.5	7.5

**Table 2:** Sensory properties of instant egusi soup mix formulation for the boiled egusi grit.

	Component Variables (g)			Response Variable ^1^					
Formulation	Egusi Grit *X*_1_	Egusi Flour *X*_2_	Egusi Hydrocolloid *X*_3_	Appearance	Colour	Aroma	Taste	Texture	Overall Acceptability
1	19.2	56.7	9.2	3.67 ± 1.03	3.50 ± 0.55	4.00 ± 1.10	3.67 ± 0.82	3.00 ± 1.10	3.50 ± 1.38
2	15.0	60.0	10.0	3.17 ± 0.98	3.33 ± 0.52	4.17 ± 0.41	3.17 ± 1.17	2.17 ± 0.41	3.50 ± 0.84
3	20.0	55.0	10.0	3.17 ± 0.75	3.33 ± 0.52	3.67 ± 1.03	3.67 ± 0.52	2.67 ± 0.82	3.50 ± 0.55
4	20.0	60	5.0	3.33 ± 0.82	3.50 ± 0.55	3.33 ± 1.03	2.83 ± 0.75	2.33 ± 1.03	2.67 ± 1.03
5	18.3	58.3	8.3	3.83 ± 0.41	3.83 ± 0.75	4.17 ± 0.75	3.83 ± 0.98	3.33 ± 1.21	3.83 ± 0.75
6	17.5	57.5	10.0	4.00 ± 0.89	3.83 ± 0.75	3.83 ± 1.33	3.67 ± 1.52	3.50 ± 1.05	3.50 ± 1.05
7	17.5	60.0	7.5	3.83 ± 0.75	3.67 ± 0.52	3.67 ± 1.03	3.83 ± 0.98	3.33 ± 1.21	3.67 ± 1.37
8	20.0	55.0	10.0	3.50 ± 0.55	3.50 ± 0.55	3.50 ± 0.84	3.33 ± 0.82	2.83 ± 1.47	3.00 ± 1.27
9	15.0	60.0	10.0	3.33 ± 1.21	3.17 ± 1.17	3.33 ± 1.21	3.83 ± 1.47	2.83 ± 1.47	3.00 ± 1.27
10	19.2	59.2	6.7	3.33 ± 1.37	3.33 ± 1.21	3.33 ± 1.37	3.33 ± 1.21	2.83 ± 1.17	3.17 ± 1.17
11	20.0	57.5	7.5	3.33 ± 0.52	3.50 ± 0.84	3.67 ± 1.21	3.17 ± 1.72	3.33 ± 1.51	3.50 ± 1.38

^1^ Values are mean ± standard deviation.

**Table 3:** Regression coefficients of a quadratic mixture model for boiled instant soup sensory quality.

Response Variables	Regression R^2^	Adjusted Regression, R^2^	Adequate Precision	Lack of Fit *p*-Value
Appearance	0.837	0.674	6.086	0.658
Colour	0.699	0.398	4.591	0.295
Aroma	0.406	−0.187	−4.623	0.761
Taste	0.899	0.799	8.554	0.924
Texture	0.712	0.463	4.676	0.619
Overall acceptability	0.721	0.442	5.670	0.876

**Table 4:** F-ratio from quadratic mixture model for instant egusi soup.

	Sensory Attribute					
Parameters	Appearance	Colour	Aroma	Taste	Texture	Overall Acceptability
Model	5.14 *	2.33	0.68	8.95 *	2.72	2.58
Linear mixture Quadratic mixture	0.05	0.37	0.71	3.87	0.34	0.93
*X*_1_ *X*_2_	17.16 *	8.35 *	0.82	7.67 *	4.58	0.98
*X*_1_ *X*_3_	7.41 *	2.14	0.27	27.91 *	4.71	6.19 *
*X*_2_ *X*_3_	0.10	0.02	0.74	0.04	2.72	3.54

* *p* ≤ 0.05: where *X*_1_ = boiled egusi grit, *X*_2_ = egusi flour: *X*_3_ = egusi hydrocolloid.

**Table 5:** Colour of instant egusi soup.

	Instant Egusi Soup ^1,2^		
Parameter	IESBG	IESSG	IESEG
L*	63.47 ± 0.16 ^a^	63.32 ± 0.33 ^a^	62.89 ± 0.05 ^b^
a*	5.77 ± 1.25 ^a^	6.00 ± 0.59 ^a^	6.34 ± 0.70 ^a^
b*	18.51 ± 1.59 ^a^	17.75 ± 0.42 ^a^	17.56 ± 1.55 ^a^

^1^ Values are mean ± standard deviation. Means with a different superscript in each row differ significantly (*p* ≤ 0.05). ^2^ IESBG = instant egusi soup boil grit, IESSG = instant egusi soup spherified grit, IESEG = instant egusi soup extruded grit.

**Table 6:** Trace mineral composition of instant egusi soup.

	Instant Egusi Soup ^1,2^		
Parameters (mg/100 g)	IESBG	IESSG	IESEG
B	2.8 ± 0.03 ^a^	3.3 ± 0.07 ^b^	3.1 ± 0.08 ^c^
Al	23.0 ± 0.28 ^a^	15.6 ± 0.18 ^b^	10.8 ± 0.16 ^c^
Ti	0.9 ± 0.02 ^a^	0.7 ± 0.02 ^b^	0.5 ± 0.01 ^c^
V	0.1 ± 0.00 ^a^	0.1 ± 0.00 ^b^	0.0 ± 0.00 ^c^
Cr	0.1 ± 0.00 ^a^	0.2 ± 0.00 ^b^	0.1 ± 0.00 ^a^
Mn	7.8 ± 0.04 ^a^	10.5 ± 0.17 ^b^	7.7 ± 0.05 ^a^
Fe	53.7 ± 0.29 ^a^	57.5 ± 0.87 ^b^	29.5 ± 0.09 ^c^
Ni	0.4 ± 0.00 ^a^	0.4 ± 0.00 ^b^	0.4 ± 0.00 ^a^
Cu	3.7 ± 0.03 ^a^	4.0 ± 0.10 ^b^	3.7 ± 0.02 ^a^
Zn	9.9 ± 0.13 ^a^	12.3 ± 0.13 ^b^	11.8 ± 0.08 ^c^
Sr	1.0 ± 0.00 ^a^	0.0 ± 0.00 ^b^	0.0 ± 0.00 ^b^
Mo	0.2 ± 0.00 ^a^	0.2 ± 0.00 ^b^	0.3 ± 0.00 ^b^
Ba	0.9 ± 0.07 ^a^	1.2 ± 0.02 ^b^	0.7 ± 0.00 ^a^
Si	17.0 ± 0.05 ^a^	13.2 ± 0.08 ^b^	11.5 ± 0.15 ^a^

^1^ Values are mean standard deviation. Means with a different superscript in each row differ significantly (*p* ≤ 0.05). ^2^ IESBG = instant egusi soup boil grit, IESSG = instant egusi soup spherified grit, IESEG = instant egusi soup extruded grit.

**Table 7:** Major mineral composition of instant egusi soup.

	Instant Egusi Soup ^1,2^		
Parameters (mg/100 g)	IESBG	IESSG	IESEG
Ca	172.3 ± 0.1 ^a^	190.9 ± 0.46 ^b^	183.4 ± 8.13 ^c^
K	1220.4 ± 26.67 ^a^	1326.2 ± 5.47 ^b^	1278.0 ± 48.79 ^a^
Mg	822.2 ± 3.36 ^a^	905 3 ± 0.83 ^b^	863.7 ± 1.97 ^c^
Na	3.9 ± 0.06 ^a^	8.8 ± 0.07 ^b^	8.9 ± 0.18 ^b^
P	1742.8 ± 16.39 ^a^	1836.3 ± 12.40 ^b^	1838.2 ± 7.51 ^c^

^1^ Values are the mean ± standard deviation. Means with a different superscript in each row differ significantly (*p* ≤ 0.05). ^2^ IESBG = instant egusi soup boil grit, IESSG = instant egusi soup spherified grit, IESEG = instant egusi soup extruded grit.

**Table 8:** Demography of panellist for instant egusi soup.

Item	Frequency (%) *
Age	
20–29	45 (90.0)
30–39	4 (8.0)
40 and above Race	1 (2.0)
Black	46 (92.0)
White	1 (2.0)
Coloured Occupation	3 (6.0)
Staff	3 (6.0)
Student Gender	47 (94.0)
Male	7 (14.0)
Female International	43 (86.0)
Yes	24 (48.0)
No	26 (52.0)

* Numbers are frequency and percentage in the bracket.

## Data Availability

All data collected is reported in this manuscript.
